# HIV-1 Diversity and Drug Resistance in Treatment-Naïve Children and Adolescents from Rio de Janeiro, Brazil

**DOI:** 10.3390/v14081761

**Published:** 2022-08-12

**Authors:** Suwellen Sardinha Dias de Azevedo, Edson Delatorre, Cibele Marina Gaido, Carlos Silva-de-Jesus, Monick Lindenmeyer Guimarães, José Carlos Couto-Fernandez, Mariza G. Morgado

**Affiliations:** 1 Laboratório de AIDS e Imunologia Molecular, Instituto Oswaldo Cruz—FIOCRUZ, Rio de Janeiro 21045-900, Brazil; 2 Centro de Ciências Exatas, Naturais e da Saúde, Departamento de Biologia—Universidade Federal do Espírito Santo—UFES, Espírito Santo 29500-000, Brazil

**Keywords:** Brazil, children, HIV-1, diversity, resistance mutations

## Abstract

The human immunodeficiency virus type 1 (HIV-1) can be transmitted via parenteral, sexual, or vertical exposure routes. The number of HIV-1 cases detected yearly in children and adolescents in Brazil did not decrease over the last decade, representing ~5% of total cases described in the country. In recent years, the HIV-1 diversity and the prevalence of transmitted drug resistance mutations (TDRM) are moving toward a marked increase. In this study, we retrospectively evaluated the diversity of HIV-1 subtypes and the TDRM prevalence in 135 treatment-naïve HIV-1 vertically infected children and adolescents born in between 1993 and 2012. These children were assessed in either 2001–2007 or 2008–2012 when they were 0 to 17 years old. The individuals assessed in 2001–2007 (*n* = 38) had median CD4+ T cell counts of 1218 cells/mm^3^ (IQR: 738–2.084) and median HIV-1 plasma viral load of 4.18 log10 copies/mL (IQR: 3.88–4.08). The individuals (*n* = 97) evaluated in 2008–2012 showed median CD4+ T cell counts of 898.5 cells/mm^3^ (IQR: 591.3–1.821) and median HIV-1 plasma viral load of 4.69 log10 copies/mL (IQR: 4.26–5.33). A steady decrease in the median CD4 T+ cell counts was observed with age progression, as expected. The majority HIV-1 *pol* sequences (87%) were classified as pure HIV-1 subtypes (77% subtype B, 9% subtype F1 and 1.5% subtype C), while 13% of sequences were classified as recombinants (CRF45_cpx, *n* = 4; CRF28/29_BF1, *n* = 2; CRF02_AG, *n* = 1; CRF40_BF1, *n* = 1, CRF99_BF1, *n* = 1, URF_BF1, *n* = 8). The overall prevalence of TDRM was 14% (19/135), conferring resistance to the nucleoside reverse transcriptase inhibitors (NRTI, 13/135–9.6%), non-nucleoside reverse transcriptase inhibitors (NNRTI, 8/135–5.9%), and protease inhibitors (PI, 2/135–1.5%). The main TDRM observed for NNRTI was the K103N (*n* = 8), while the mutations T215I/Y/D/E (*n* = 7) and M184V (*n* = 4) were the main TDRM for NRTI. Only two TDRM were observed for PI in one individual each (M46I and V82A). Most TDRM were found in the HIV-1 subtype B (84%) sequences. This study reveals an HIV-1 epidemic with high diversity and moderate prevalence of TDRM in the pediatric population of Rio de Janeiro, indicating the existence of possible problems in the clinical management of prophylactic therapy to prevent mother-to-child transmission and future treatment options for the affected children.

## 1. Introduction

The human immunodeficiency virus type 1 (HIV-1) can be transmitted via parenteral, sexual, or vertical exposure routes [1]. The risk of HIV-1 mother-to-child transmission (MTCT) can be reduced from ~40% to <2% levels using prenatal antiretroviral therapy (ART) [2,3,4] and is associated with the maternal viral load (VL) decline. The HIV-1 prevalence among pregnant women in Brazil is low (<1%) [5]; however, in the last 20 years the HIV-1 MTCT rate reported in Brazil has been varying in different regions [6,7,8,9] of the country, reaching high levels (1.6 to 9%) when compared to the recommended Pan American Health Organization goal (<2%) for achieving the elimination of MTCT of HIV. During this same period, almost 40% of pregnant women diagnosed with HIV in Brazil lived in the Southeast region, 10% of them in Rio de Janeiro [10]. Currently, more than 80% of HIV-infected pregnant women in the country have had access to antiretroviral therapy [11]. The number of HIV cases detected yearly in children and adolescents (newborns until nineteen years-old) in Brazil did not decrease over the last decade, representing ~5% of the total cases [10]. The same trend is observed in the AIDS incidence rate among children and adolescents in Rio de Janeiro that remain stable, between 2–3%, and are mostly concentrated in the metropolitan region.

The Brazilian Public Health system adopts universal and free access to ART as prophylaxis to prevent HIV MTCT and for mother and child treatment [12]. However, if the child is vertically infected with a drug-resistant HIV-1 strain, the first-line ART regimens may be compromised, which increases the odds of virologic failure. The number of studies evaluating the prevalence of transmitted drug resistance mutations (TDRM) in the Brazilian pediatric population, particularly from Rio de Janeiro state, is very scarce. Most TDRM rates estimated in children from Brazil indicate moderate prevalence levels (~10%), however, the rates vary greatly among studies (ranging from 0% to 16.2%), according to the considered period and/or region [13,14,15,16,17,18,19]. In Rio de Janeiro, the only study addressing TDRM in children found no evidence of resistance in a pediatric cohort enrolled between 1999 and 2003 [20]; however, this work had serious limitations in properly assessing the prevalence of TDRM due to the limited number of samples. A recent survey on acute/recent HIV infected individuals from Rio de Janeiro described an overall TDRM prevalence of 16.3% [21]

HIV-1 subtype-specific natural amino acid background and viral genetic polymorphisms may play a role in the development of resistance to antiretroviral drugs [22,23]. In Brazil, the HIV-1 molecular epidemic is mostly driven by the subtypes B (~70%), C (1–5%), F1 (5–10%), and recombinants among them (~20%) [24]. The HIV molecular epidemiology among pregnant women in Rio de Janeiro resembles the one found in the country [25,26,27]. However, the molecular profile seems to be moving towards a marked increase in diversity, as other HIV-1 clades and recombinants have been regularly found in recent years, and hence reducing the prevalence of subtype B [28,29]. Several transmission networks comprising non-B HIV-1 subtypes were described in the Rio de Janeiro state, comprising the subtype D [30], CRF02_AG [31], and CRF45_cpx [32] strains, some of them isolated from children.

Studies conducted by our group described an increase in the TDRM rate (from 10.7% to 17.2%) and in the prevalence of non-B HIV-1 subtypes (from 11% to 32%) between 2005–2015 among HIV-1-infected pregnant women from Rio de Janeiro [25,26]; thus, we hypothesize that a similar trend might have occurred in the HIV-1-infected pediatric population from Rio de Janeiro. According to this scenario, we retrospectively evaluated the prevalence of TDRM and the viral diversity in 135 treatment-naïve HIV-1 vertically infected children and adolescents born between 1993 and 2012, which were assessed in either 2001–2007 or 2008–2012 when they were 0 to 17 years old.

## 2. Materials and Methods

### 2.1. Study Subjects

We retrospectively evaluated the HIV-1 *pol* (protease/reverse transcriptase-PR/RT) sequences obtained from treatment-naïve HIV-1-positive vertically infected children and adolescents attended in clinics of the Public Health System in the state of Rio de Janeiro. In this study, we analyzed a convenience sampling of 135 HIV-1 *pol* PR/RT sequences from the database stored at AIDS and Immunology Laboratory/FIOCRUZ. Two sampling periods were compared: (1) 2001–2007, HIV-1 *pol* sequences obtained from 38 HIV-1 infected children under two years old born to HIV-1 positive women. The children’s blood samples were collected for HIV diagnosis and HIV-1 *pol* genotyping for TDRM surveillance; and (2) 2008–2012, 97 HIV-1 *pol* sequences from children and adolescents (aged 0–17 years old) submitted to HIV genotypic drug resistance testing as part of the standard procedures of the Brazilian Network for HIV-1 Genotyping (RENAGENO). The samples assessed in 2008–2012 were further subdivided into four different groups according to the age at the sampling: <5 years (*n* = 63); 5–9 years (*n* = 22); 10–14 years (*n* = 10); and 15–17 years (*n* = 2). The differences observed in the age distribution over the two periods partially reflect changes in the Brazilian public policies for carrying out HIV-1 genotyping tests in the pediatric population.

Information on sex, age, municipality of origin, last viral load quantification, and CD4+ T lymphocyte counts were retrieved from the genotyping test form when available. There was no discerning information between intrauterine, at delivery, or breastfeeding transmission. Samples were geographically grouped into four regions based in the Brazilian Institute of Geography and Statistic (https://www.ibge.gov.br/geociencias/organizacao-do-territorio/divisao-regional accessed on 1 September 2018) procedures: (1) Metropolitan region I (includes the Rio de Janeiro capital and neighboring municipalities); (2) Metropolitan region II (comprising the municipalities from the Baixada Fluminense area, an impoverished area in the outskirts of Rio de Janeiro’s capital); (3) Coastal Region; and (4) Northern region. This study was approved by the Oswaldo Cruz Institute Ethics Committee (CAAE 03925112.0.0000.5248).

### 2.2. Extraction, PCR Amplification and Sequencing of HIV-1 RNA

In the 2001–2007 period, the entire protease (PR) and reverse transcriptase (RT) gene sequences were obtained from plasma samples from HIV-1 positive individuals using the ViroSeq™ HIV Genotyping System (version 2.0; Applied Biosystems, Foster City, CA, USA), under conditions recommended by the manufacturers. The purified PCR products were sequenced with an ABI PRISM 3100xl Genetic Analyzer (Applied Biosystems, Foster City, CA, USA). In the 2008–2012 period, HIV-1 PR and RT nucleotide sequences were generated using the TRUGENE^®^ HIV-1 Genotyping Assay (Siemens HealthCare Diagnostics, Tarrytown, NY, USA), following the recommendations of the manufacturers. The ViroSeq HIV-1 kit covers the entire PR-coding region and the first 320 amino acids of RT. The TRUGENE HIV-1 sequences span the PR (amino acids 4 to 99)- and RT (amino acids 40 to 240)-coding regions. The laboratory is VQA (Virology Quality Assessment) certified for HIV genotyping using both methods, which guarantees the quality of the sequences obtained throughout the study period.

### 2.3. Transmitted Drug-Resistance Mutation (TDRM) Analyses

Sequences were evaluated for the presence of mutations suggestive of TDR with the Calibrated Population Resistance (CPR) Tool Version 8.0 [33] using 2009 Surveillance Drug Resistance Mutation list [34] available on the Stanford HIV Drug Resistance Database (https://hivdb.stanford.edu/cpr, accessed on 1 March 2022). To predict the effect of the identified TDRM on drug susceptibility, sequences bearing mutations identified by the CPR algorithm were classified as susceptible, low-level resistant, intermediate level resistant, or high-level resistant to the drug classes and specific drugs using the Stanford HIVdb Program, version 9.0 (Stanford University, Palo Alto, CA, USA).

### 2.4. HIV-1 Subtypes Classification and Recombination Analyses

Preliminary HIV-1 subtype classification was made with REGA subtyping tool v3.46 [35] and was intended to identify putative pure subtypes, circulating, and unique recombinant forms (CRFs and URFs, respectively). This classification was confirmed by phylogenetic analyzes after the alignment of the new 135 HIV-1 *pol* PR/RT sequences (covering HXB2 nucleotide coordinates 2253 to 3290) with all known HIV-1 group M subtypes and CRFs reference sequences gathered from Los Alamos HIV Sequence Database (http://www.hiv.lanl.gov, accessed on 1 March 2022), using ClustalW algorithm implemented in MEGA v7 program [36]. All reference sequences from pure subtypes and CRFs indicated by REGA and those classically described in South America were included in the phylogenetic analysis (Supplementary Table S1). Drug resistance mutation sites were retained in the final alignment.

Phylogenetic trees were built using Maximum Likelihood (ML) method implemented in the program PhyML v3.0 [37]. The nucleotide substitution model used was the GTR+I+G, selected by the jModeltest program [38]. Heuristic tree search was performed using the SPR branch-swapping algorithm and the reliability of the topology obtained was estimated with the approximate likelihood-ratio test (aLRT) based on the Shimodaira–Hasegawa-like procedure [39]. Only clusters with aLRT values above 0.8 were considered significant. Trees were evaluated in the Figtree software (http://tree.bio.ed.ac.uk/software/figtree, accessed on 1 March 2022). The sequences classified as recombinant forms were further evaluated on Simplot software v3.5.1 [40] to confirm the CRF-like and URF profiles. Bootstrap values supporting branching with HIV-1 reference sequences were determined by NJ trees constructed using the K2–parameter substitution model, based on 100 resamplings, with a 300-nucleotide sliding window moving in steps of 10 bases.

### 2.5. HIV-1 Reference Datasets and Phylogenetic Analyses for Subtype B, CRF02_AG and URF_BF1

HIV-1 subtype B *pol* PR/RT sequences from this study were aligned with subtype B reference sequences representative of the B_PANDEMIC_ (*n* = 300) and the B_CAR_ (*n* = 200) clades described previously [41,42]. The HIV-1 *pol* PR/RT sequence from this study classified as CRF02_AG was aligned with CRF02_AG reference sequences, respectively, from Africa and Brazil available on Los Alamos HIV Database. The CRF02_AG clustering pattern followed the criteria described previously [31]. The non-CRF-like recombinant sequences identified in this study were aligned with all URF BF1 Brazilian recombinants available on Los Alamos HIV Database. The clustering pattern was investigated by performing ML phylogenetic analyses as described above, and only those clusters encompassing the recombinant sequences from this study and their close relative Brazilian sequences that branched until the second ancestral node in the ML phylogenetic tree with aLRT values above 0.8 were considered significant.

### 2.6. Statistical Analysis

All statistical analyses were done with GraphPad Prism version 6.0 (GraphPad Software Inc., La Jolla, CA, USA) using Fisher’s exact test, one-way and two-way ANOVA with Tukey’s multiple comparisons tests. A *p*-value less than 0.05 was considered statistically significant.

### 2.7. Sequence Data

All the partial HIV-1 *pol* sequences generated in this study were evaluated by the HIV-1 sequence quality tool from Los Alamos HIV sequence database (https://www.hiv.lanl.gov/content/sequence/QC/index.html, accessed on 1 October 2021) to ensure that only sequences with high quality were used in this study and submitted to the NCBI GenBank database. The GenBank database accession numbers for the HIV-1 *pol* sequences described in this study are OL624883-OL625013.

## 3. Results

### 3.1. Epidemiological, Clinical, and Virological Characteristics of HIV-1 Infected Children and Adolescents

One hundred thirty-five treatment-naïve HIV-1 infected children and adolescents were evaluated during the timeframe of this study (2001 to 2012). The limited clinical and epidemiological data available for the 38 HIV-1 infected children assessed in the 2001–2007 period is provided in Supplementary Table S2. All subjects were less than two years-old at the time of sampling, with a median age of six months (Interquartile range [IQR]: 3–12 months), and no information about the individuals’ sex was available. Most individuals sampled (35/38, 92%) were from the Metropolitan region I, while 8% (3/38) were from the Metropolitan region II. No AIDS-related conditions were reported in the children assessed at the 2001–2007 period, who exhibited a median CD4+ T cell counts of 1218 cells/mm^3^ (IQR: 738–2,084 cells/mm^3^) and plasma HIV-1 viral load median of 4.18 log10 copies/mL (IQR: 3.88–4.08 log10 copies/mL).

Ninety-seven children and adolescents were included in this study from 2008 to 2012. The main clinical and epidemiological characteristics of these individuals are shown in Table 1.

Most subjects (65%) were younger than five-years-old when the genotyping test was conducted (median: 1 year, IQR: 0.91–2 years). The number of individuals in each age group decreased with age progression, reaching 23% in the age group 5–9 years old (median: 6 years, IQR: 5–8 years) and 12% in the age group 10–17 years old (median: 11 years, IQR: 10.75–13.25 years). In general, the pediatric population evaluated in the 2008–2012 period was comprised of 53% females, comprising most individuals sampled in the <5 and 10–17 years old age groups (52% and 83%, respectively). The frequency of male individuals was slightly higher (54%) only in the 5–9 years age group (Table 1); however, there was no statistically significant differences between the sexes (*p* = 0.4078).

The municipality of origin was available from almost all (*n* = 97) individuals sampled in 2008–2012, and they were distributed as follows: the Metropolitan region I comprised most samples (56%, 54/97), followed by Metropolitan region II (40%, 37/97), Northern region (3%, 4/97) and Coastal region (l%, 1/97). However, these geographical differences were not statistically significant (*p* = 0.0621, Table 1). 

The overall median CD4+ T cell counts before genotyping was of 898.5 cells/mm^3^ (IQR: 591.3–1.821 cells/mm^3^) and only four individuals presented CD4+ T cell counts <100 cells/mm^3^. However, we observed a steady decrease in the median CD4+ T cell counts with age progression, since these individuals were not on ART (Table 1, *p* < 0.0001). The overall median HIV-1 plasma viral load observed in this period was of 4.69 log10 copies/mL (IQR: 4.26–5.33 log10 copies/mL). The highest median plasma HIV-1 viral load (5.02 log10 copies/mL, IQR: 4.38–5.63 log10 copies/mL) was observed in the youngest group (<5 years, *p* = 0.0011), in which the maximum limit of detection (>500,000 copies/mL) was observed in 14 individuals. In the remaining groups, the median plasma HIV-1 viral load was 4.57 log10 copies/mL (IQR: 3.85–4.85 log10 copies/mL) in the 5–9 years and 4.17 log10 copies/mL (IQR: 3.48–4.44 log10 copies/mL) in the 10–17 years (Table 1).

### 3.2. Analysis of HIV-1 Subtypes and Recombination Pattern

**Figure 1:**
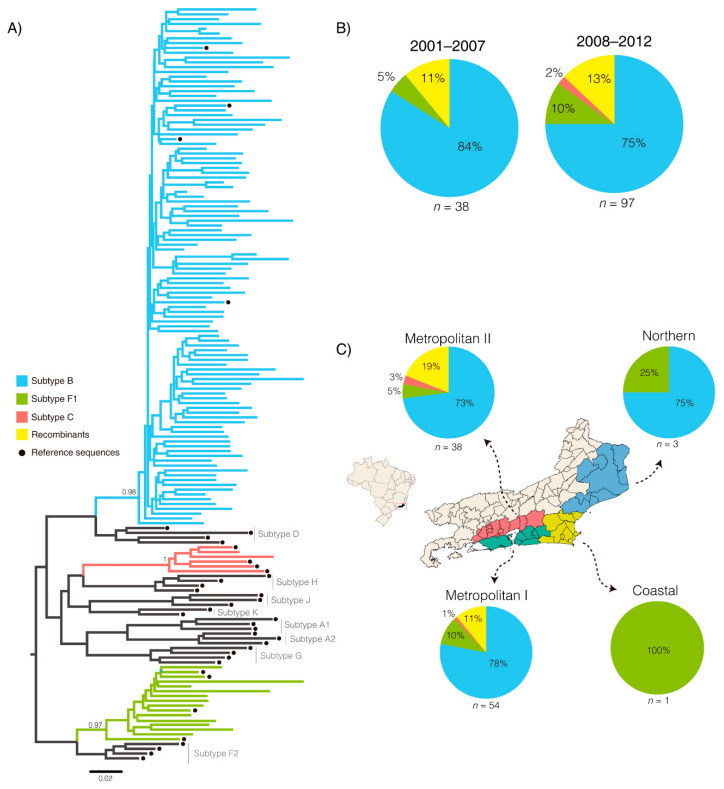
Subtype classification of the *pol* (PR/RT) region of HIV-1 from infected children and adolescents from Rio de Janeiro. (**A**) Maximum likelihood tree of the HIV-1 strains classified as “pure” subtypes. Reference sequences retrieved from the Los Alamos HIV-1 data base were indicated by black circles. The branches were colored according to the legend at left. aLRT values are shown only at key nodes. The scale represents number of substitutions per site. (**B**) Pie charts representing HIV-1 molecular diversity found in the two time periods. (**C**) Map of Rio de Janeiro state colored according to different regions indicating the local HIV-1 diversity.

The phylogenetic analysis of the HIV-1 *pol* PR/RT regions of 135 treatment-naïve children and adolescents showed that most sequences (87%, 118/135) were classified as pure subtypes, followed by 13% (17/135) of sequences exhibiting patterns of recombination. Among the HIV-1 *pol* sequences classified as pure subtype in this genomic region (Figure 1A), the most prevalent HIV-1 clade was subtype B (77%, 104/135), followed by subtype F1 (9%, 12/135) and subtype C (1.5%, 2/135). All HIV-1 *pol* sequences classified as subtype B detected in this study branched with high support (aLRT = 0.95) within the B_PANDEMIC_ clade (Supplementary Figure S1).

In the 2001–2007-time interval, 89% (34/38) of the HIV-1 *pol* sequences were classified as pure subtypes, and subtype B was the most prevalent HIV-1 clade (84%, 32/38) followed by subtype F1 (5%, 2/38). The recombinant forms were observed in 11% (4/38) of the HIV-1 *pol* sequences in this period (Figure 1B). In the most recent period (2008–2012), 87% (84/97) of HIV-1 *pol* sequences were classified as pure subtypes, of which 75% (72/97) were classified as subtype B while subtypes F1 and C exhibited frequencies of 10% (10/97) and 2% (2/97), respectively (Figure 1C). Recombinant forms were observed in 13% (13/97) HIV-1 *pol* sequences. No statistically significant differences were observed when comparing the molecular diversities between the two periods (*p* = 0.2410, even when only children <5 years old were considered in the 2008–2012 period (*p* = 0.0872). The highest viral diversity was observed in the metropolitan regions I and II, with predominance of subtype B (≥78%), and minor circulation of subtypes F1 (≥10%) and C (≥3%), and recombinant forms (≥19%) (Figure 1C). The other regions showed more restricted HIV-1 diversity with circulation of only subtypes B and F1.

**Figure 2:**
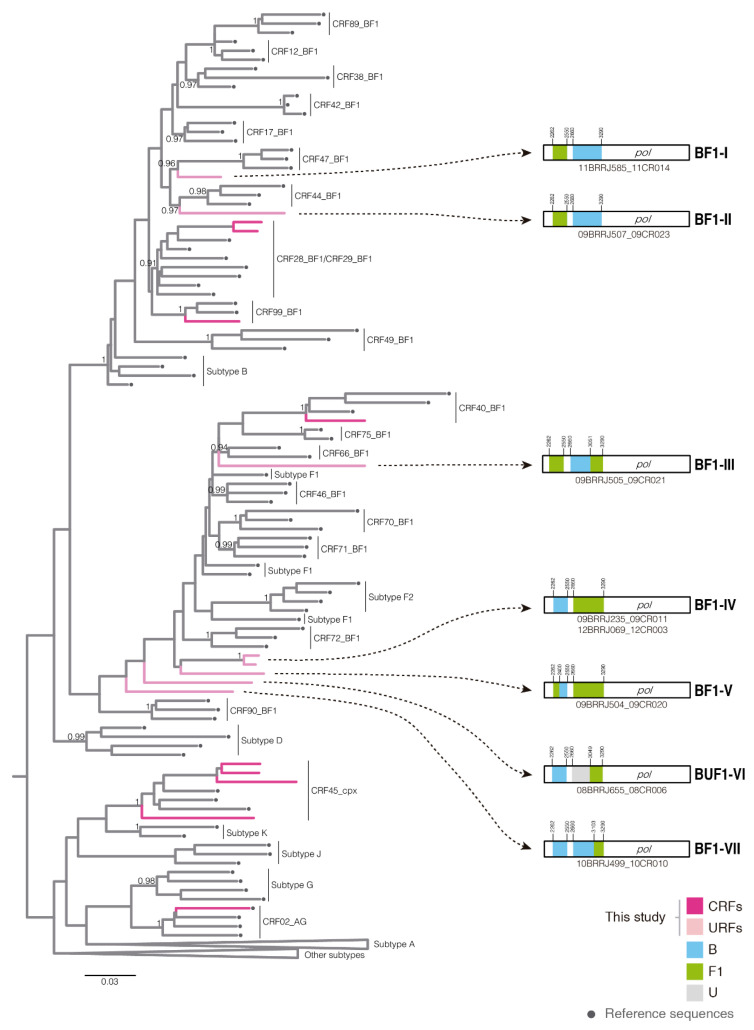
Maximum likelihood tree of the pol (PR/RT) region of HIV-1 infected children and adolescents from Rio de Janeiro classified as recombinants. Gray circles indicate reference sequences of all CRFs included. CRFs clusters were indicated by gray vertical lines. Some branches were collapsed for visual clarity. The branches of the sequences classified as CRFs were colored dark pink while those classified as URFs were colored light pink. The aLRT values are shown at key nodes and the scale represents number of substitutions per site. Schematic drawing showing breakpoint pattern at the pol (PR/RT) region of each URF lineage were showed in the right, using the color scheme indicated in the legend. The rectangles represent the whole pol gene, while the colored boxes indicate the position of the PR/RT sequences obtained in this study and the genomic positions (relative to the HXB2 reference sequence) of the recombination breakpoints.

Most recombinant strains (6%, 8/135) displayed a BF1 mosaic structure and were distributed into seven independent lineages, of which five comprised only one sequence each, while one lineage comprised two sequences and all of them were classified as URF_BF. Two BF1 sequences displayed the same mosaic structure and branched together with the CRF28/29_BF (*n* = 2, aLRT = 1) and was classified as CRF28/29_BF1-like. One sequence branched with CRF40_BF1 (aLRT = 1) and other with CRF99_BF1 (aLRT = 1) reference sequences, being thus classified as CRF40_BF1-like and CRF99_BF1-like recombinants. One sequence displayed a BUF1 mosaic structure and was further classified as an URF_BUF1. We also identified one AG recombinant (0.6%, 1/135) and four AUK recombinants (3%, 4/135) that branched with high support with CRF02_AG (aLRT = 1) and CRF45_cpx (aLRT = 0.97) reference sequences, respectively, and were classified as CRF02_AG-like and CRF45_cpx-like recombinants (Figure 2).

The CRF02_AG strain identified in this study clustered together with high support (aLRT = 0.86) inside a previously described CRF02_AG Brazilian cluster (02_AGBR-II) comprising other strains from Rio de Janeiro (Supplementary Figure S2).

All non-CRF_BF1-like recombinant sequences (6%, 8/135) identified in this study were combined with all Brazilian URF_BF1 sequences available in the Los Alamos HIV Database (Supplementary Figure S3). The ML phylogenetic analyses showed that lineages BF1-I and BF1-V were not related to other Brazilian URFs. By contrast, the lineage BF1-II branched with high support (aLRT = 0.96) with three URF_BF1 strains from Rio de Janeiro and one from São Paulo, while the lineage BF1-III branched with highest support (aLRT = 1) with two BF1 sequences from Rio de Janeiro. The BF1-VI and BF1-VII lineages branched (aLRT 0.85 and 0.99, respectively) with one Brazilian BF1 sequence from Rio de Janeiro, each. The lineage BF1-IV, comprising two sequences from this study, did not branch with other Brazilian BF sequences (Supplementary Figure S3).

### 3.3. Transmitted Drug Resistant Mutations in HIV-1 Children and Adolescents

According to the CPR analysis, the overall TDRM prevalence in the 2001–2007-time interval was 24% (9/38). Table 2 summarizes nucleoside reverse transcriptase inhibitors (NRTIs), non-nucleoside reverse transcriptase inhibitors (NNRTIs) and protease inhibitors (PI) transmitted drug resistance mutations, HIV-1 subtype and resistance profiles level. All TDRM observed in this period conferred resistance exclusively to reverse transcriptase inhibitors. Mutations conferring resistance to NRTIs were present in 21% (8/38) of the strains, with higher prevalence of the T215I/Y (11%, 4/38) and M184V (8%, 3/38) mutations. The only NNRTI mutation was K103N, found in 8% (3/38) of the sequences. The M184V mutation confers high HIV-1 resistance to lamivudine (3TC) and emtricitabine (FTC), in addition to low resistance to abacavir (ABC), whereas the K103N mutation confer high resistance to efavirenz (EFV) and nevirapine (NVP). The mutations T215I or M41L alone confer low resistance to zidovudine (AZT) while M41L and T215Y combined confer low resistance to tenofovir (TDF), intermediate resistance to ABC and high resistance to AZT. The mutation P225H confers intermediate resistance to doravirine (DOR). Most mutations were found in the sequences classified as subtype B (89%, 8/9).

In the 2008–2012 period, the overall TDRM prevalence was 10% (10/97) (Table 2). In this period, TDRM conferring resistance to NRTIs and NNRTIs exhibited the same frequencies (5% each, 5/10) while TDRM for PI were observed in two sequences (2%, 2/97). Three different aminoacidic substitutions were observed at codon 215 in three strains (3%, 3/97). These mutations confer low resistance to AZT. Another sequence showed low resistance to AZT due to the presence of the F77L mutation. The M184V mutation, which confers low resistance to ABC and high resistance to FTC and 3TC, was found in one sequence. The mutations observed for PI were M46I—which conferred low resistance to nelfinavir (NFV), indinavir (IDV), lopinavir (LPV), atazanavir (ATV)–, and V82A conferring low resistance to ATV and intermediate resistance to IDV, LPV, and NFV. Most TDR mutations found in 2008–2012 were present in sequences classified as subtype B (80%, 8/10).

Despite the apparent reduction in TDRM prevalence observed between the two periods (from 24% to 10%), there was no statistical significance (*p* = 0.2365). The reduction in TDRM prevalence between 2001 and 2012 remained similar (from 24% to 9.5%) when only children <5 years old were considered in the 2008–2012 period; however, this difference was not statistically significant (*p* = 0.1278).

## 4. Discussion

This study shows a complex epidemic scenario amongst treatment-naïve HIV-1 infected children from the Rio de Janeiro Metropolitan Region, which seems to have persisted for more than a decade, between 2001 and 2012. We observed the co-circulation of pure HIV-1 subtypes and diverse and rare recombinant forms, including CRF02_AG and CRF45_cpx, coupled with a reduction in the prevalence of TDRM from 2001–2007 to the 2008–2012 period.

HIV infection in children is challenging, mainly due to the immaturity status of the immune response [43]. Disease progression in vertically infected children shows mainly two key distinct patterns: early progression, with the median age of symptom onset at 4 months of age; or late progression, with a median age of symptom onset at 6 years old [43,44,45]. A meta-analysis evaluating CD4+ T cells levels and viral load in children as predictors of disease progression [46] found that children older than two years old had an increased risk of progression to AIDS when the CD4+ T cell percentages were below 15% and when the viral load exceeded 10^5^ copies per mL [46]. In our study, regardless of the period analyzed, most individuals had CD4+ T cell levels above 500 cells/mm^3^, with a gradual decrease observed in the older groups. This was expected since the number of CD4 cells in children gradually decrease with age progression until approach to adult values [47]. Until 2007, the viral load levels were slightly lower than those observed in the 2008–2012 period, with median viral loads reaching the highest levels (10^5^ viral copies per mL) in children under five years. According to the PENTA guidelines [48], ART should be started urgently in all infants irrespective of clinical or immunological stage. In children aged > 12 months with no or minor symptoms (CDC clinical stage A or N or WHO stage 1 or 2), ART should be started when the CD4+ T cell count falls below 1000 cells/mm^3^ for children aged 1 to 3 years or when the CD4+ T cell count falls below 500 cells/mm^3^ for children aged 3 to 5 years. In children >5 years, the treatment should start when the CD4+ T cell count falls below 350 cells/mm^3^.

In 2011, the World Health Organization defined criteria for the Global Plan and eliminate pediatric HIV infections in the world by 2015 [49]. The proposed targets range from the completion of at least one prenatal consultation and an HIV diagnosis for 95% of pregnant women during prenatal care and the immediate initiation of antiretroviral therapy for 90% of HIV-infected pregnant women. The Brazilian government acts actively to reduce the HIV-1 vertical transmission by offering free prenatal and delivery coverage, as well as ensuring universal and free access to ART, HIV follow-up screenings, and breastmilk substitutes. Therefore, the follow-up of the antiretroviral naïve pediatric population tends to become increasingly rare. The presence of ARV-resistant HIV strains in untreated individuals may occur in the setting of broad ARV coverage due to factors such as poor adherence to treatment regimens, combined with the lower genetic barrier of some drugs [50]. These variants that carry antiretroviral resistance mutations may limit the future availability of therapeutic options in newly infected individuals, especially in children who acquired the infection perinatally, increasing the risk of virological failure.

In this study, we found different scenarios regarding the TDRM prevalence in the two time periods analyzed: in the 2001–2007 period, the overall TDRM prevalence was high (24%), reducing to moderate levels (10%) in the 2008–2012 period. The TDRM reported in the Brazilian pediatric population is historically low, ranging from 0% to 12.8% [13,18,19,20,51]. One study from Manaus—Brazil, on the other hand, reported a TDRM prevalence of 16.2% in children, indicating that the TDRM prevalence scenario in Brazil may be more complex than previously thought [17]. The high TDRM prevalence found in this study in the 2001–2007 period could be partially explained by the therapy adopted in Brazil until 2007 to prevent MTCT. The preventive MTCT standard of care was initially based on the monotherapy with zidovudine, which was later modified to a dual-therapy regimen based on transcriptase inhibitors [12,52]. TDRM conferring resistance to AZT (M41L, F77L, T215I/Y/E/D) were found in 13% of the individuals assessed in 2001–2007, decreasing to 4% in the 2008–2012 sampling period. The zidovudine monotherapy for HIV-1 MTCT prevention can lead to a selection of minority resistant HIV-1 populations that can be transmitted during childbirth [53]. Furthermore, zidovudine monotherapy and a higher maternal viral load were already described as significantly associated with transmission [4]. The moderate TDRM levels observed in this pediatric population since 2008 reflect the advantages of adopting triple antiretroviral therapy, comprising two NRTIs and one NRTI as the initial regimen for children [12]. The moderate TDRM found was lower than a study conducted in Rio de Janeiro including ART-naïve patients with acute/recent HIV infection that found an overall TDRM prevalence of 16.3% [21].

A previous study associated the presence of any DRM in the mother with the presence of any DRMs in infants, supporting the recommendation that all pregnant women and HIV-infected infants undergo HIV genotypic resistance testing prior to initiation of ART [54]. In our study, regardless of the period evaluated, 19 children (14%) had some TDRM. Of these, 89% had viral strains resistant to reverse transcriptase inhibitor drugs, with 53% conferring mutations exclusively for NRTI, 21% only for NNRTI, and 16% for both. Contrasting with our results, in a study conducted on newly diagnosed untreated HIV-infected children from Rio de Janeiro sampled between 2000 and 2003, no TDRM was found. The authors suggested that as most of the children were the index case in the family, the perinatal transmission likely occurred from individuals not aware of their HIV status, and then, no prophylaxis therapy was adopted [20]. Only two individuals had TDRM for NNRTI+PI and PI. Both the M184V and the T215I mutations were the most frequent for the NRTI class. M184V is selected in therapeutic regimens that include the antiretroviral drugs 3TC/FCT and reduces susceptibility to these by up to 100-fold [55]. Furthermore, it gives a low level of resistance to ABC, while it increases susceptibility to drugs such as AZT and TDF [34]. T215I is a thymidine analog mutation (TAM) that may confer a low level of resistance to AZT [55]. For the NNRTI class, the most frequent mutation was K103N, which is known to be selected in individuals who received treatment with NVP or EFV [34]. This mutation confers high resistance to these two drugs. The prevalence of TDRM against NNRTI class, mainly K103N mutation, still poses an important threat to the ART response (virologic clearance) in the HIV-1-infected population from Rio de Janeiro in recent times [21].

The historical context is important to understanding the dynamics of the molecular diversity changing in the HIV-1 epidemics. In Brazil, several studies reported HIV-1 epidemics dominated by subtypes B, F1, C, and the recombinants among them, with a recent trend in the increase of diversity [28,29]. In this context, our study represents an important background to understanding the HIV-1 diversity of the Rio de Janeiro state in the heterosexual population, since the vertically infected children represent a proxy of the heterosexual route of transmission. Contrary to expected, there was no significant difference in HIV-1 diversity between the two periods, indicating that the HIV epidemic profile in the pediatric population of Rio de Janeiro exhibits a remarkable prevalence of non-B subtypes and recombinant forms since the beginning of the 2000s. It is important to note that the finding observed here likely reflect transmission events occurring years before sampling, since the children in our pediatric cohort were born between 1993 and 2012, and probably got infected via MTCT. In this regard, previous studies conducted with pregnant women in Rio de Janeiro found a significant increase in the HIV-1 non-B clades from 2005 to 2015 [25,26], and our results indicate that the change in the subtype B prevalence in Rio de Janeiro probably started several years before.

All subtype B sequences identified in this study clustered within the B_PANDEMIC_ clade, confirming that this clade accounts for almost all subtype B infections in the Rio de Janeiro state [56]. The absence of subtype C in the 2001–2007 period and its low prevalence in the 2008–2012 period indicates that the sampling period is prior to the influx of this clade from the Brazilian southern region [57]. Some unique recombinant forms found in this study showed similar mosaic structures to other sequences from Brazil. Two putative new CRFs carrying the subtypes B and F1 were found, comprising isolates from Rio de Janeiro and São Paulo states. More studies are necessary to determine whether these recombinant sequences represent new CRFs or are simply URF displaying the same recombination pattern in the *pol* region. Besides the HIV-1 pure subtypes and CRFs classically found in the HIV-1 epidemic in Brazil [29], we also found one CRF02_AG and four CRF45_cpx sequences. The CRF02_AG strain branched within a cluster that has spread locally in Rio de Janeiro state over the last 30 years [31,57]. The four CRF45_cpx strains identified in this study were previously described as participating in an autochthonous transmission network that has spread to the Rio de Janeiro, São Paulo, and Minas Gerais states [32]. The high molecular diversity found in the HIV-1-infected pediatric population indicates that the Rio de Janeiro state has acted as a hub for the introduction and spread of new HIV-1 clades in the Brazilian HIV epidemic since the 2000s.

The main limitation of this study was the distinct age of the populations sampled in the two periods. These differences could introduce a bias since some TDRM could revert in older prenatally ART-naïve infected children [58], resulting in an underestimation of their prevalence. It is important to point out that all children included in this study were ART drug-naïve at the moment of inclusion and the majority (65%) of the children included in the 2008–2012 period were <5 years. The prevalence of TDRM was similar in the overall sample of the 2008–2012 period (10/97) compared with the subsample (6/63) of children <5 years (10% versus 9.5%, respectively). However, caution is warranted when comparing this study with others since it represents an epidemiological scenario from more than ten years ago. In that period, the universal access to ART began to be expanded in Brazil, with the offer of new pediatric therapeutic options that facilitated the initiation of global goals for the end of the AIDS epidemic by 2030 (90-90-90) established in 2014 by UNAIDS.

## 5. Conclusions

In summary, the results presented here indicate an important decrease in the TDRM prevalence in the pediatric population in Rio de Janeiro between 2001–2007 and 2008–2012 while no differences were observed in the HIV-1 molecular diversity among these two periods. The TDRM found may limit the future therapeutic options in the children infected perinatally, increasing the risk of virological failure. Our study also found high molecular diversity circulating in Rio de Janeiro since the beginning of the 2000s, with the presence of HIV-1 clades rarely detected in Brazil. More studies are necessary to evaluate the TDR prevalence in HIV-infected children in more recent periods, especially considering pretreatment genotyping assessment to determine the optimal ART regimen to be used.

## Figures and Tables

**Table 1:** Virological, demographic, and laboratory data of HIV-infected children analyzed between 2008 and 2012.

Parameter	Age Groups			*p*-Value
	<5 (*n* = 63)	5–9 (*n* = 22)	10–17 (*n* = 12)	
CD4+ T cell count (cells/mm^3^)	1298 (738–2084)	813 (374–1028)	570 (234–637)	<0.0001 *
Plasma HIV RNA load (log10 RNA copies/mL)	5.0 (4.4–5.6)	4.6 (3.8–4.8)	4.2 (3.5–4.5)	0.0011 *
**Sex, no. (%)**				
Female	33 (52)	10 (46)	10 (83)	0.4078 **
Male	30 (48)	12 (54)	2 (17)	
**Geographical origin, no. (%)**				
Metropolitan region I	36 (57)	12 (54)	6 (50)	0.0621 **
Metropolitan region II	23 (36)	9 (41)	5 (42)	
Northern region	3 (5)	1 (5)	-	
Coastal region	1 (2)	-	-	
Unknown	-	-	1 (8)	

Values are expressed as median (25th–75th IQR) or number of cases (percentage in parentheses). * One-way ANOVA. ** Two-way ANOVA.

**Table 2:** Transmitted drug resistance mutations and resistance profiles according to time of sampling.

Period	Sample ID	Age	Subtype	NRTI Mutations	NNRTI Mutations	PI Mutations	Resistance Profiles			
							Low	Intermediate	High	
2001–2007	PC_01	<2	B	T215I	-	-	AZT	-	-	
	PC_15	<2	B	-	K103N	-	-	-	EFV, NVP	
	PC_19	<2	B	M41L, T215Y	K103N	-	TDF	ABC	AZT, EFV, NVP	
	PC_21	<2	B	M184V	K103N, P225H	-	ABC	DOR	FTC, 3TC, EFV, NVP	
	PC_25	<2	B	M184V	-	-	ABC	-	3TC, FTC	
	PC_27	<2	B	M184V	-	-	ABC	-	3TC, FTC	
	PC_32	<2	B	M41L	-	-	AZT	-	-	
	PC_36	<2	B	T215I	-	-	AZT	-	-	
	PC_38	<2	45_cpx	T215I	-	-	AZT	-	-	
2008–2012	09CR012	<2	B	F77L	-	-	AZT	-	-	
	09CR023	14	URF_BF	-	K103N	-	-	-	EFV, NVP	
	10CR009	11	B	-	-	M46I	NFV, ATV, IDV, LPV	-	-	
	10CR013	<2	B	T215I	-	-	AZT	-	-	
	10CR019	<2	B	M184V	K103N, Y181C	-	ABC, DOR	ETR, RPV	FTC, 3TC, EFV, NVP	
	11CR012	9	B	T215E	-	-	AZT	-	-	
	11CR016	3	C	-	K103N	-	-	-	EFV, NVP	
	12CR005	<2	B	-	K103N	-	-	-	EFV, NVP	
	12CR006	6	B	T215D	-	-	AZT	-	-	
	12CR013	<2	B	-	K103N	V82A	ATV	IDV, LPV, NFV	EFV, NVP	

NFV—nelfinavir; ABC—abacavir; 3TC—lamivudine; AZT—zidovudine; DOR—doravirine; EFV—efavirenz; ETR—etravirine; FTC—emtricitabine; NVP—nevirapine; IDV—indinavir; LPV—lopinavir; ATV—atazanavir; RPV—rilpivirine.

## Data Availability

The GenBank database accession numbers for the HIV-1 *pol* sequences described in this study are OL624883-OL625013.

## Supplementary Materials

The following supporting information can be downloaded at: https://www.mdpi.com/article/10.3390/v14081761/s1, Supplementary Table S1. HIV-1 pol reference sequences used in the subtype classification and recombination analysis. Supplementary Table S2: Main clinical information of the children sampled between 2001–2007. Supplementary Figure S1: Maximum-likelihood phylogenetic tree for HIV-1 B subtype classification of the pediatric sequences from Rio de Janeiro; Supplementary Figure S2: Maximum-likelihood phylogenetic tree of HIV-1 CRF02_AG *pol* sequences from Brazil and African countries; Supplementary Figure S3: Maximum-likelihood phylogenetic trees of HIV-1 URFs *pol* sequences from Brazil.
