# Supplementary material for: HIV-1 Diversity and Drug Resistance in Treatment-Naïve Children and Adolescents from Rio de Janeiro, Brazil
Source: Viruses. 2022 Aug 12;14(8):1761. doi: 10.3390/v14081761 (PMC9413768; doi:10.3390/v14081761)
Supplement: Supplementary file 1 [file viruses-14-01761-s001.zip › viruses-1676460-supplementary.pdf]

viruses

Supplementary Materials

HIV-1 Diversity and Drug Resistance in Treatment-Naïve Children and Adolescents from Rio de Janeiro, Brazil

Suwellen Sardinha Dias de Azevedo [1, †, *], Edson Delatorre [2, †], Cibele Marina Gaido [1], Carlos Silva-de-Jesus [1], Monick Lindenmeyer Guimarães [1], José Carlos Couto-Fernandez [1] and Mariza G. Morgado [1]

[1] Laboratório de AIDS e Imunologia Molecular, Instituto Oswaldo Cruz — FIOCRUZ, 21045-900, Rio de Janeiro, Brazil.

[2] Centro de Ciências Exatas, Naturais e da Saúde, Departamento de Biologia —Universidade Federal do Espírito Santo—UFES, 29500-000, Espírito Santo, Brazil.

† These authors contributed equally to this work

* **Correspondence:** suwellen@ioc.fiocruz.br or suwellendias@gmail.com

Supplementary Table S1. HIV-1 *pol* reference sequences used in the subtype classification and recombination analysis

Subtype or CRF	Accession numbers
A1	DQ676872, AB253421, AB253429
A2	AF286238, GU201516, AF286237
A3	AY521629, AY521631, AY521630
A4	AM000053, AM000054
A6	EU861977, AF413987, KU749403
A7	KX389622, KX389608, MH078558
A8	MW353966, MW353967
B	K03455, AY423387, AY173951, AY331295
C	U52953, U46016, AF067155, AY772699
D	K03454, AY371157, AY253311, U88824
F1	AF077336, AF005494, AF075703, AJ249238
F2	AY371158, AJ249236, AJ249237, AF377956
G	AF084936, AF061641, U88826, AY612637
H	AF190127, AF190128, AF005496, FJ711703
J	EF614151, GU237072, AF082394
K	AJ249235, AJ249239
L	MN271384, AF286236, AF457101
02_AG	AY271690, AB485636, L39106
12_BF	AF408629, AF408630, AF385936
17_BF1	EU581825, EU581827, EU581828
28_BF1	DQ085872, DQ085873, DQ085874
29_BF1	DQ085876, JF804807, AY455778, DQ085871
31_BC	EF091932, AY727526, AY727527
38_BF1	FJ213781, FJ213782, FJ213780
39_BF1	EU735534, EU735536, EU735535
40_BF1	EU735538, EU735540, EU735539
42_BF1	EU170155, EU170147, EU170153
44_BF1	FJ358521, AY536235, MH078557
45_cpx	FN392874, FN392876, FN392877
46_BF1	DQ358801, DQ358802, HM026456
47_BF1	KJ849798, GQ372987, FJ670529
66_BF1	MK298150, JN251903
70_BF1	KJ849758, KJ849775, KU749388
71_BF1	KJ849761, KJ849762, KJ849773
72_BF1	KJ671534, KJ671535, KJ671536
75_BF1	GU595149, GU595160
81_cpx	MH986016, MH986017
89_BF1	AY037271, KX818199, KX818200
90_BF1	KY628219, KY628223, KY628221
99_BF1	MH986014, MH986013

Supplementary Table S2. Main clinical information of the children sampled between 2001-2007

Children (n=38)	
Age (months)	6 (IQR: 3-12)
Geographical origin	
Metropolitan I	92% (n=35)
Metropolitan II	8% (n=3)
Clinical information	
$CD4^+$ cell count (cells/mm3)	1.218 (IQR: 591-1753)
Viral load (log_{10} copies/mL)	4.18 (IQR: 3.87- 4.08)

Values are expressed as median (25th–75th IQR) or number of cases (percentage in parentheses).

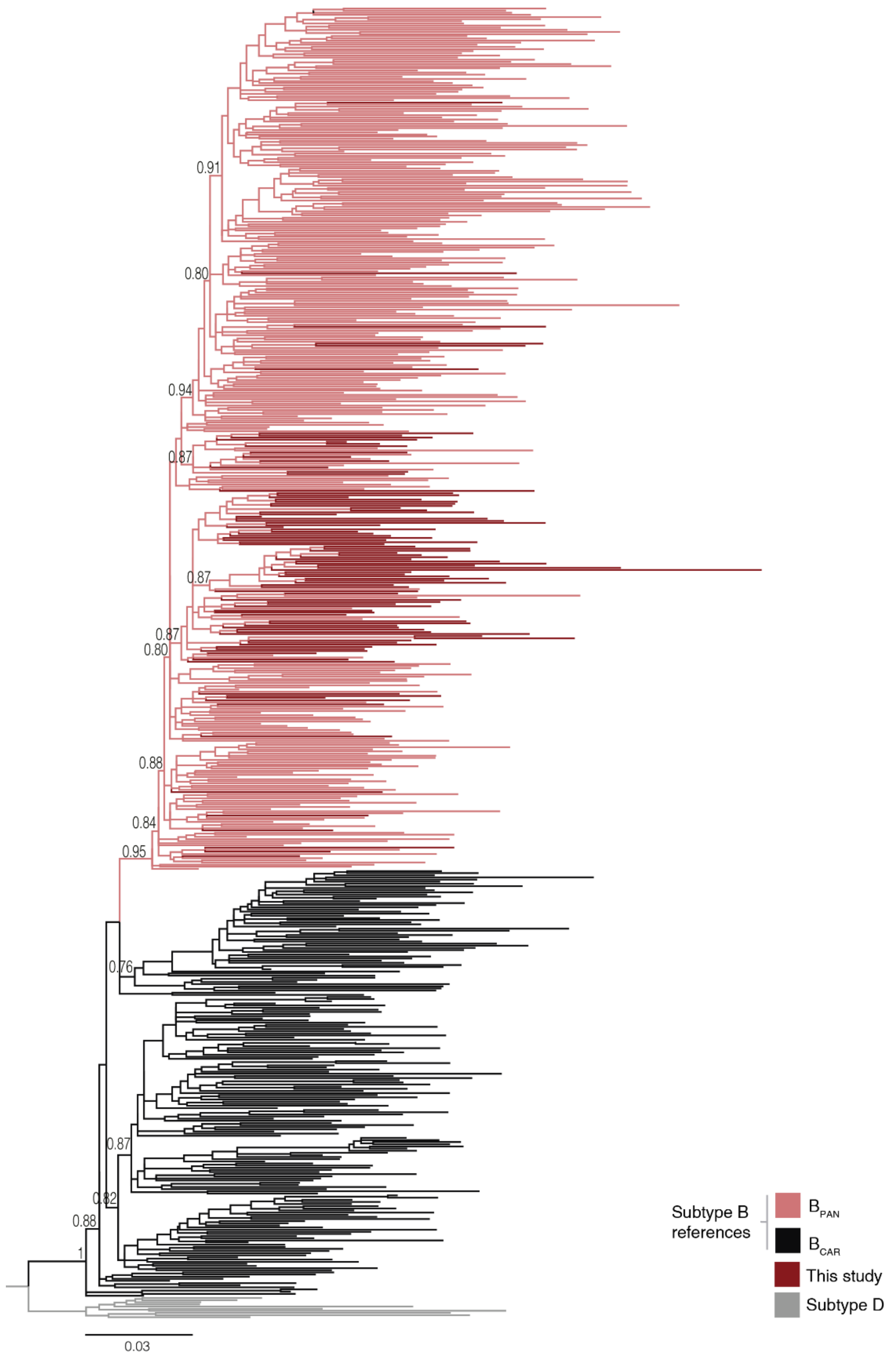

Supplementary Figure S1. Maximum-likelihood phylogenetic tree for HIV-1 B subtype classification of the pediatric sequences from Rio de Janeiro. References sequences of the subtype B Caribbean (BCAR) and Pandemic (BPAN) clades are colored black and pink, respectively. Subtype B sequences from this study are represented by red branches. The aLRT branch support values are indicated at key nodes. The subtype D sequences (gray) were used to root the tree. Branch lengths are drawn to scale with the bar at the bottom indicating nucleotide substitutions per site.

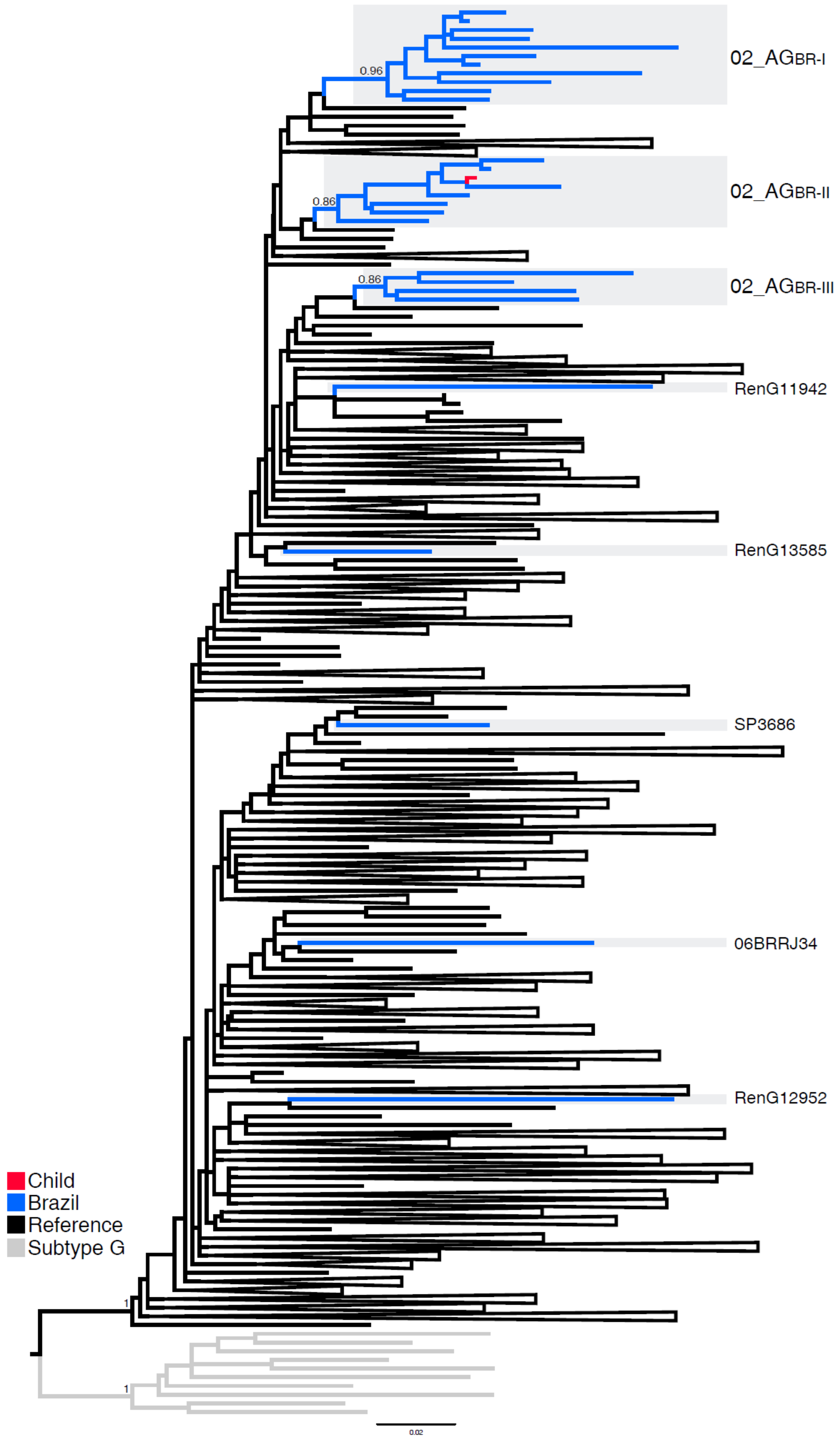

Supplementary Figure S2. Maximum-likelihood phylogenetic tree of HIV-1 CRF02_AG *pol* sequences from Brazil and African countries. Sequences from Africa are colored black, while sequences from Brazil and from this study are represented by blue and red branches, respectively. The grey shaded boxes indicate the Brazilian CRF02_AG lineages. The aLRT support values are indicated only at key nodes. HIV-1 subtype G sequences (gray) were used to root the tree. Horizontal branch lengths are drawn to scale with the bar at the bottom indicating nucleotide substitutions per site.

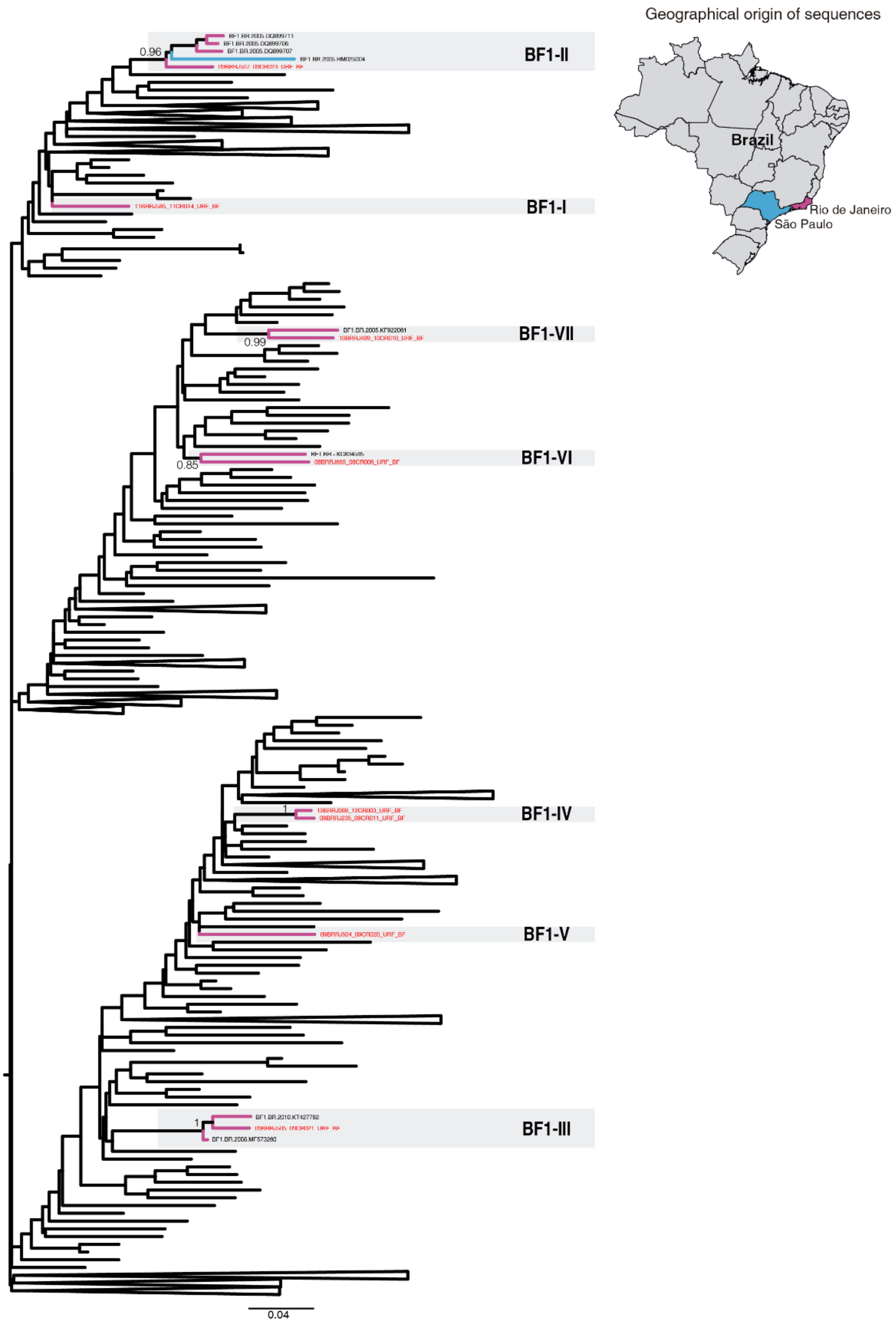

Supplementary Figure S3. Maximum-likelihood phylogenetic trees of HIV-1 URFs *pol* sequences from Brazil. URF_BF1 sequences from this study are indicated by an asterisk and were combined with all URFs_BF1 Brazilian recombinants available at Los Alamos HIV Database. Branches are colored according to the Brazilian state of sampling as indicated in the map. For visual clarity, some clades are collapsed into triangles and horizontal branch lengths are drawn to scale with the bar at the bottom indicating nucleotide substitutions per site.
